# What’s in a name? Characteristics of clinical biofilms

**DOI:** 10.1093/femsre/fuad050

**Published:** 2023-09-01

**Authors:** Mads Lichtenberg, Tom Coenye, Matthew R Parsek, Thomas Bjarnsholt, Tim Holm Jakobsen

**Affiliations:** Costerton Biofilm Center, Department of Immunology and Microbiology, University of Copenhagen, Blegdamsvej 3B, 2200 Copenhagen, Denmark; Laboratory of Pharmaceutical Microbiology, Ghent University, Ottergemsesteenweg 460, 9000 Ghent, Belgium; Department of Microbiology, University of Washington School of Medicine, 1705 NE Pacific St., WA 98195 Seattle, United States; Costerton Biofilm Center, Department of Immunology and Microbiology, University of Copenhagen, Blegdamsvej 3B, 2200 Copenhagen, Denmark; Department of Clinical Microbiology, Copenhagen University Hospital, Ole Maaløes vej 26, 2100 Copenhagen, Denmark; Costerton Biofilm Center, Department of Immunology and Microbiology, University of Copenhagen, Blegdamsvej 3B, 2200 Copenhagen, Denmark

**Keywords:** aggregates, gene expression, infection, microcolonies, microenvironment, phenotypic

## Abstract

*In vitro* biofilms are communities of microbes with unique features compared to individual cells. Biofilms are commonly characterized by physical traits like size, adhesion, and a matrix made of extracellular substances. They display distinct phenotypic features, such as metabolic activity and antibiotic tolerance. However, the relative importance of these traits depends on the environment and bacterial species. Various mechanisms enable biofilm-associated bacteria to withstand antibiotics, including physical barriers, physiological adaptations, and changes in gene expression. Gene expression profiles in biofilms differ from individual cells but, there is little consensus among studies and so far, a ‘biofilm signature transcriptome’ has not been recognized. Additionally, the spatial and temporal variability within biofilms varies greatly depending on the system or environment. Despite all these variable conditions, which produce very diverse structures, they are all noted as biofilms. We discuss that clinical biofilms may differ from those grown in laboratories and found in the environment and discuss whether the characteristics that are commonly used to define and characterize biofilms have been shown in infectious biofilms. We emphasize that there is a need for a comprehensive understanding of the specific traits that are used to define bacteria in infections as clinical biofilms.

## Teaser

*In vitro* biofilms are microbial communities that differ from individual cells by displaying distinct physical features, phenotypic traits, and antibiotic tolerance, but clinical biofilms may not possess the same characteristics and must be better understood for accurate definition and characterization in clinical settings.

## Introduction

Historically, biofilms have been characterized by various features that distinguish them from planktonic populations. The first descriptions of biofilms were based on their morphological properties, as tools for visible observations were the only methods available until a few decades ago. The term ‘*biofilm*’ was used for the first time in a publication from 1981 (McCoy et al. 1981). Before introducing the term ‘biofilm’, several studies have described the phenomenon of bacteria making clumps or small microcolonies. In the 1930s, some of the first detailed descriptions of microbial attachment to glass surfaces submerged in water were published. They observed growing cells on the surface forming microcolonies increasing in size and described the organisms to grow in ‘a fairly uniform film’ (Henrici 1933, Zobell and Allen 1935). The first reported clinical observation of what we today recognize as biofilms were presented in 1977. A Gram-stained smear of a sputum sample from a cystic fibrosis (CF) patient revealed ‘heaps of bacteria’ (Hoiby 1977). However, clumps of bacteria had already been reported in the 1650s by van Leeuwenhoek and were also mentioned in a publication from 1883 that described how bacteria grow on a surface to form clumps, i.e. ‘biofilms’ (Weismann et al. 2007). The visual inspection of biofilms entered a new era after the introduction of confocal laser scanning microscopy, which allowed investigation of the formation of *in vitro* grown bacterial communities in greater detail (Lawrence et al. 1991). The subsequent introduction of various molecular methods allowed a more holistic approach to characterize biofilms. For example, staining of specific exopolysaccharides has revealed the existence of self-produced matrix components (Cowan et al. 2000, Sohm et al. 2011). Further, the introduction of various system level approaches made it possible to characterize genomic, transcriptomic, proteomic, and metabolomic differences between microorganisms living different lifestyles. As we continue to investigate environmental and clinical systems, it will be important to know if small clusters or groups of cells (that are commonly observed in these samples) are exhibiting biofilm-like properties and physiology. There have been many descriptions and discussions regarding the definition of biofilms (Costerton et al. 1999, Sauer et al. 2022) and the main goal of this review is not to establish a new definition of a biofilm, but rather to discuss the various characteristics, alone or in combination, that can be used to define a biofilm.

## How to diagnose a biofilm?

Biofilms can exhibit great diversity depending on their species, composition, and local environment. Factors such as nutrient availability, pH, temperature, and the presence of multiple organisms all have an impact on the structure and composition of a biofilm within a single species. As a result, the characteristics of a biofilm can vary greatly between different ecosystems. Thus, under certain conditions biofilms are intricate and highly dynamic communities of microorganisms that interact with each other and with their surroundings, adapting to create complex structures (Flemming and Wuertz 2019). These communities are extremely resilient and can endure harsh conditions. As a consequence, biofilms can survive and thrive in a variety of environments and can range in size from a few microns to several millimeters in thickness (Reysenbach and Cady 2001, Bjarnsholt et al. 2013) and can be enclosed in a matrix consisting of extracellular polymeric substance (EPS) that can promote individual cells to stick together, adhere to surfaces, and provide protection from environmental stressors (Wingender et al. 1999).

**Figure 1.**
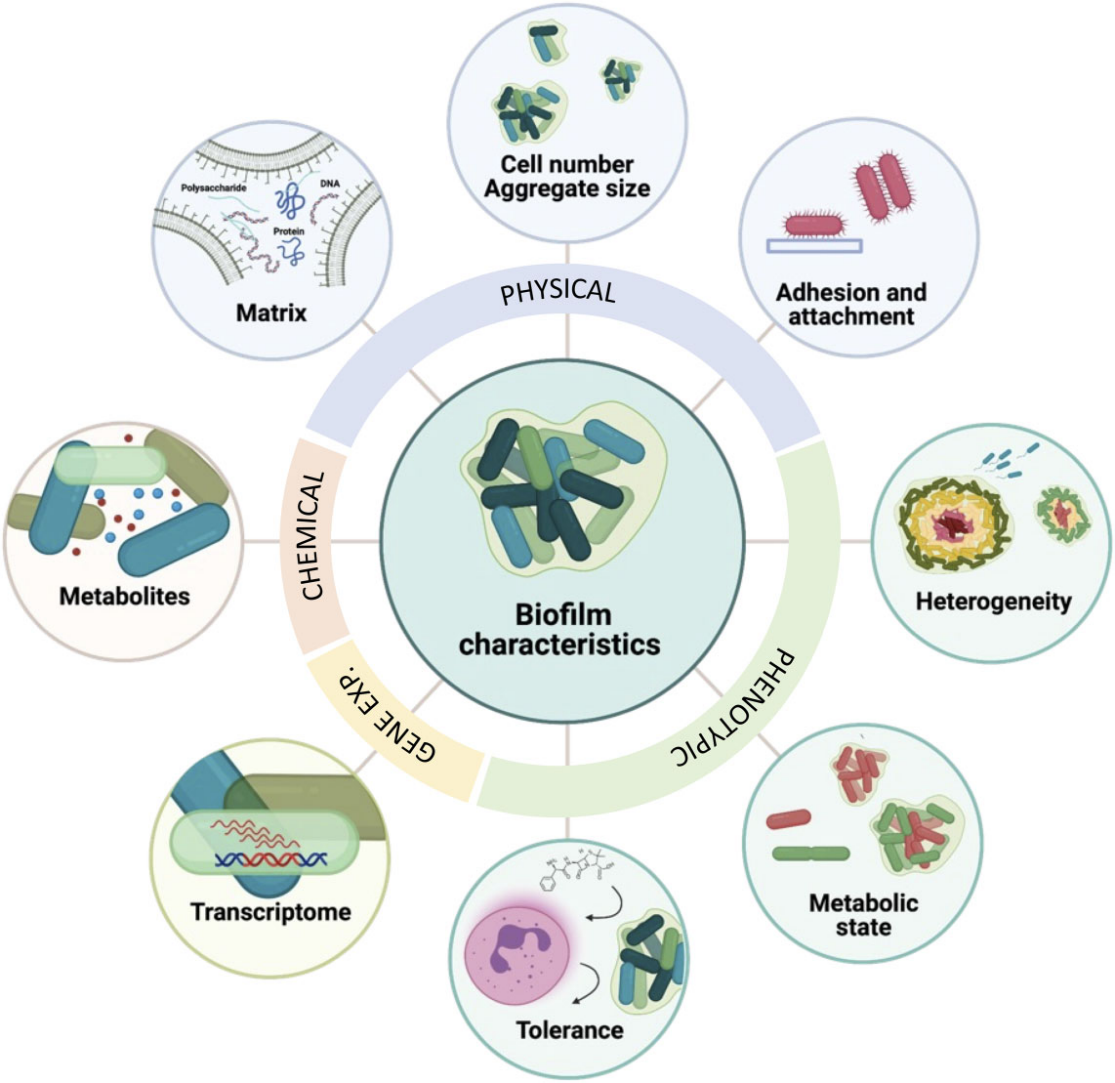
Characteristics that are commonly used to define biofilms include the number of cells attached to a surface and/or present in aggregates, attachment factors, presence of (heterogeneous) (sub)populations and physicochemical gradients, tolerance to antibiotics and external stressors, cell-to-cell communication, altered gene expression, metabolically distinct phenotypes, and the presence of self-produced extracellular matrix.

Not surprisingly, there are obvious differences between *in vivo* biofilms (i.e. those occurring in a clinical setting and in the natural or man-made environment), and *in vitro* biofilms grown in the laboratory (Hall-Stoodley et al. 2004), but even *in vitro* biofilms can be diverse in terms of phenotype and architecture (Pamp and Tolker-Nielsen 2007, McBain 2009). This diversity highlights the importance of a discussion on which characteristics can be considered hallmarks of a biofilm. The various characteristics shown in Fig. 1 will serve as the foundation for this review. We divided the central aspects that can be used to characterize a biofilm into the following four features, (i) physical, (ii) chemical, (iii) phenotypic, and (iv) gene expression profiles. This review will include findings of biofilms from various settings, but the primary focus will be on clinical biofilms.

## Physical features

The physical features of bacterial biofilms are complex and varied and can play an important role in the survival and persistence of bacteria in diverse environments. The three key physical features traditionally used to describe a biofilm are (i) a community of cells in close proximity, (ii) adhesion/attachment of cells to a biotic or abiotic surface, and (iii) aggregates encased in a self-produced or externally provided matrix. These characteristics vary greatly depending on the environment and the microorganisms involved, resulting in a diverse range of biofilm sizes and structures. If one were to take this approach to define a biofilm, an obvious question is whether a cellular aggregate must be a certain size or contain a minimum number of cells before it can be characterized as a biofilm? For example, can we define two cells embedded in a matrix as a biofilm? Or conversely, can we define thousands–millions of cells in close contact as a biofilm even if they are not embedded in an obvious matrix?

### Physical size

Biofilms have been found in a broad size range in infections ranging from large multicellular aggregates to small clusters of only a few µm in diameter (Bjarnsholt et al. 2013). The physical dimensions of aggregates is increasingly recognized as an important factor as it influences the development of physiological heterogeneity within biofilms (Stewart and Franklin 2008). The dynamics underlying the observed size distribution are not clear, but they are influenced by multiple parameters such as access to metabolic substrates, grazing by predators or immune cells, antimicrobial compounds, physical constraints, and so on. In theory, there is no upper limit of biofilm size but in many soft tissue infections, biofilms are typically found in the range from 5 to 200 µm (Bjarnsholt et al. 2013). In the environment, biofilms such as the photosynthetic mats commonly found in hot springs can easily be observed by the naked eye. While there is no consensus on a specific threshold for the number of cells required to form a biofilm, many articles have used a lower diameter of 5 µm to distinguish biofilms from single cells (Bay et al. 2018, Kolpen et al. 2022). In a recent study, it was further shown that successful phagocytosis of bacterial aggregates by polymorphonuclear leucocytes (PMNs) dramatically decreased with aggregate diameters of >5 µm (Alhede et al. 2020b, Pettygrove et al. 2021).

Thus, a selective pressure may act on biofilms to attain a certain size to resist such competition. However, such selective pressures are complex and can vary depending on the specific environmental conditions and species involved. It thus appears that the size and structure of biofilms can be influenced by a combination of genetic factors, environmental cues, and microbial interactions within the community and with their surroundings.

### Matrix

Self-produced EPS, or the biofilm matrix, remains one of the most common characteristics used in definitions of biofilms. The bacterial EPS consists of a range of different biopolymers such as polysaccharides, proteins, and DNA and its function, composition, and diversity have been thoroughly reviewed elsewhere (Flemming et al. 2023).

Production of self-produced matrix has been demonstrated in e.g. CF sputum (Jennings et al. 2021) and in chronic wounds (Kirketerp et al. 2008). However, is a self-produced matrix a necessity to define an aggregate of cells as a biofilm? It has, e.g. been shown that several species rely on the matrix production of other species to form biofilm (Chenicheri et al. 2017). In airway infections, bacteria are found in aggregates embedded in host mucus and it has been shown that host-derived eDNA surrounds aggregates of bacteria effectively shielding them from their surroundings (Alhede et al. 2020a). In wounds, bacteria can be immobilized in necrotic tissue and wound slough (Kirketerp-Moller et al. 2008) and in many other soft tissue infections, host secretions have been found to contain bacteria (Bjarnsholt et al. 2013).

Interestingly, a recent study showed a multitude of single, spatially separated, bacterial cells in secretions from a range of acute and chronic pulmonary infections (Kolpen et al. 2022). The study showed the presence of polysaccharides within the biofilms by Alcian Blue staining but the finding questions whether the conventional ‘biofilm’ mode of growth and self-produced EPS is necessary for surviving a persistent inflammatory response. Finally, Jennings et al. (2021) recently demonstrated that self-produced matrix is produced and surrounds *Pseudomonas aeruginosa* aggregates from CF sputum. The infectious microenvironment is often characterized by being high in nutrients, but oxygen depleted, thus creating a limit for the metabolic rate (Bjarnsholt et al. 2022, Lichtenberg et al. 2022a), which may hinder production of EPS as this production is associated with elevated metabolic expenditure (Lichtenberg et al. 2022a).

### Aggregation and adhesion mechanisms

A multitude of different mechanisms of biofilm formation have been elucidated through decades of research and an expansion of the biofilm life cycle was recently proposed to include both attached and nonattached biofilms (Sauer et al. 2022). Initial surface colonization by bacteria is achieved through active adhesion (via e.g. type IV pili) and followed by clonal growth and potential recruitment of other bacteria that can ‘stick’ to the matrix. For nonattached biofilms, three mechanisms are currently known: (i) restricted motility whereby clonal expansion will create aggregated bacteria, (ii) bridging aggregation where bacteria stick to each other by production of EPS, and (iii) depletion aggregation where aggregates can be enclosed by polymers by entropic forces in certain environments (Kragh et al. 2023).

These mechanisms describe how attached or nonattached clusters of bacteria can form. If the mechanism can be identified from a given cluster of cells, this can be used to infer other information about the bacterial community, e.g. certain gene expression patterns are correlated with some of the mechanisms. For example, in Gram-negative species an increased levels of c-di-GMP is associated with matrix production (Andersen et al. 2021). However, none of these mechanisms can infer extensive information on the behaviour or phenotypical traits of the bacteria in the biofilm, other than that they, at some point, formed a biofilm. Additionally, these mechanisms do not explain the occurrence of slow growing, spatially separated, single cells in inflamed host secretions (Kolpen et al. 2022).

## Gene expression profiles

There is an ever-growing number of studies in which gene expression is compared between planktonic (suspended) microbial cells and biofilm-associated cells (Whiteley et al. 2001, Schembri et al. 2003, Dotsch et al. 2012, 2015, Alio et al. 2020, Zheng et al. 2022, Wang et al. 2022b, Toliopoulos and Giaouris 2023). In almost all of these studies differences in expression levels are observed for a smaller or larger fraction of genes, although comparisons between different studies are difficult at best, due to differences in experimental conditions (different model systems for biofilm and planktonic growth, temperature, growth media, duration of biofilm formation, and so on) and as a consequence there is very little overlap between genes identified as up- or downregulated in biofilms in different studies (Coenye 2010). In addition, many studies are limited by the low accuracy of the laboratory models used, and the transcriptomic profiles obtained from *in vitro* or nonhuman *in vivo* models, may differ substantially from the transcriptome during human infection, as was, e.g. shown for *P. aeruginosa* (Cornforth et al. 2018, 2020, Harrington et al. 2022, Lewin et al. 2023) and *Staphylococcus aureus* (Xu et al. 2016, Ibberson and Whiteley 2019, Le Masters et al. 2021).

In addition, microbial biofilms are not homogeneous populations (Lenz et al. 2008) and as a consequence gene expression data obtained from such populations by definition present an ‘average picture’, that may not necessarily reflect meaningful biological signals. Early studies on heterogeneity in biofilms required generating mutants in which gene expression could be monitored microscopically (e.g. by creating GFP transcriptional fusions) (Ito et al. 2009), a combination of isolating single cells and qPCR (Perez-Osorio et al. 2010), or isolating subpopulations, followed by transcriptional profiling with microarrays (Williamson et al. 2012, Heacock-Kang et al. 2017). More recently, probe hybridization-based approaches have been used to map spatial differences in bacterial biofilms (Dar et al. 2021, Livingston et al. 2022). While these approaches differ in resolution and throughput, they all confirm that spatially resolved heterogeneity is the norm, not the exception, highlighting the importance of the (physicochemical) microenvironment in shaping the microbial transcriptome and phenotype (Dar et al. 2021, Bjarnsholt et al. 2022, Lichtenberg et al. 2022a). This heterogeneity is not only observed in biofilms, but also in planktonic cultures (Lenz et al. 2008, Ryall et al. 2012). For example, it was found that up to 90% of the biomass of *P. aeruginosa* ‘planktonic’ cultures consists of cellular aggregates with a diameter of 10–400 µm (Schleheck et al. 2009). Recent technological advances have made it possible to perform single-cell RNA (scRNA) sequencing on bacterial cells and pioneering scRNA-seq studies have confirmed heterogeneity in various planktonic bacterial populations, including *Bacillus subtilis, Salmonella enterica, Escherichia coli*, and *Clostridium perfringens* grown in various rich media (Brennan and Rosenthal 2021, Kuchina et al. 2021, Homberger et al. 2023, McNulty et al. 2023). Various innovative scRNA-seq approaches hold great promise for the future investigation of heterogeneity of microbial populations, both sessile and planktonic, and especially approaches that allow to link specific expression profiles with spatial information and/or information about the physicochemical microenvironment will yield novel insights (Wang et al. 2023).

Variability between strains from one species or multiple closely related species should also be considered. In *P. aeruginosa*, variability in transcriptional profiles between 77 clinical strains was higher when these were grown as biofilms than when they were grown planktonically, suggesting the impact of the genetic background of individual strains on which genes are expressed in biofilms is bigger than the impact on which genes are expressed in planktonic cultures (Thoming et al. 2020). The core biofilm transcriptome (i.e. genes differentially expressed between planktonic and sessile cultures in all 77 clinical *P. aeruginosa* isolates) consisted of only 143 genes, 103 that were commonly upregulated in biofilms and 30 commonly downregulated compared to planktonic cultures. Among the upregulated genes were several genes required for pyoverdine biosynthesis, heme assimilation, and central carbon metabolism, as well as genes encoding superoxide dismutase and fumarate hydratase. Downregulated core genes include genes involved in denitrification and aerobic arginine catabolism (Thoming et al. 2020). Among the top 250 biofilm-expressed genes in seven *Stenotrophomonas maltophilia* isolates, 106 genes were commonly expressed in all isolates, while 142 of the 250 most strongly expressed genes were only expressed in one of seven isolates (Alio et al. 2020). Notably, the expression of the majority of these 250 genes strongly expressed in *S. maltophilia* biofilms is not biofilm-specific, as they are also highly expressed in planktonic cultures. In *S. aureus*, profound differences were observed in biofilm-associated gene expression in representatives of three important MRSA clones (Vlaeminck et al. 2022). When comparing expression differences between planktonic and sessile populations at the KEGG pathway level, the number of pathways varied from 11 (*S. aureus* ST239), over 27 (*S. aureus* USA300) to 58 (*S. aureus* HEMRSA-15). Moreover, only a single common differentially expressed gene was identified across these three *S. aureus* clones, i.e. *clfA*, encoding clumping factor A (Vlaeminck et al. 2022). Interstrain heterogeneity in gene expression was also observed in *Salmonella* Typhimurium (Zheng et al. 2022) and *Listeria monocytogenes* (Toliopoulos and Giaouris 2023) biofilms.

While most studies have focused on differences between planktonic and sessile cultures, it is worth mentioning that based on transcriptomic analyses, dispersed *P. aeruginosa* cells (i.e. cells released from a biofilm) are different from both planktonic and sessile cells, and that the mode of dispersion has a profound influence on gene expression in dispersed cells (Chua et al. 2014, Wille et al. 2020).

The currently available data seem to indicate that there is no such thing as a universal ‘biofilm transcriptome’, nor is there any evidence for a universal ‘planktonic transcriptome’ or ‘dispersed cell transcriptome’. An important reason for this is the heterogeneity commonly found in microbial populations; these populations more resemble a collection of subpopulations with distinct properties, rather than a collection of cells with identical properties. With further technical advances in transcriptome analysis and imaging, it will likely become feasible to determine spatial differences in gene expression in microbial biofilms at the single-cell level. This may shed more light on the relationship between the microenvironment, local differences in gene expression, and phenotype.

## Chemical features

From a spatial perspective, the distribution of e.g. metabolites may be used to characterize biofilms. In planktonic cultures, a homogenous distribution will be expected whereas biofilms will produce heterogeneous landscapes of metabolite concentration due to reaction–diffusion processes (Stewart 2003, Pabst et al. 2016, Stewart et al. 2016, 2019, Kirketerp-Møller et al. 2020).

Are certain metabolic products always present in biofilms? Often e.g. active denitrification or fermentation is used to exemplify that oxygen has been consumed by dense biofilm structures (Pabst et al. 2016). However, the expression of anaerobic metabolic pathways is not biofilm specific.

There are only few studies investigating the proteome of biofilms by proteomics and/or metabolomics. A recent study used targeted and untargeted metabolomics to compare the metabolism of biofilm and planktonic cultures of the clinical uropathogenic *E. coli* UTI 89 strain. A metabolic reprogramming was found to be involved in biofilm formation by increasing metabolites, such as amino acids, sugars, lipids, uridines, and organic acids that are essential for EPS synthesis (Lu et al. 2019). The metals Fe^3+^, Mn^2+^, and Mg^2+^ have been reported to regulate biofilm formation by regulation of functional metabolism in *E. coli* (Guo and Lu 2020, Guo et al. 2021, Wang et al. 2022a).

The nucleotide second messengers cAMP and bis-(3′–5′)-cyclic dimeric GMP (c-di-GMP) are involved in biofilm formation. High intracellular levels of c-di-GMP are associated with formation of a biofilm, while low levels are associated with the planktonic lifestyle (Hengge 2009, Dahlstrom and O’Toole 2017, Collins et al. 2020, Martinez-Mendez et al. 2021). In general, the expression and/or activity of flagella is reduced by high levels of c-di-GMP whereas the expression of adhesins and biofilm-associated exopolysaccharides is upregulated. In *P. aeruginosa*, c-di-GMP positively regulates the production of several matrix components (alginate, CdrA adhesin, Cup fimbriae, and Pel/Psl polysaccharides) (Borlee et al. 2010, Baraquet and Harwood 2013, Fazli et al. 2014). Opposite to c-di-GMP, the global transcription factor cAMP receptor protein (CRP) can both promote and inhibit biofilm formation. As an example, CRP promote biofilm formation in *E. coli* and *P. aeruginosa*, whereas it inhibits biofilm formation in *Serratia marcescens* and *Vibrio cholerae* (Liu et al. 2020). In addition, it modulates biofilm maintenance in *Shewanella putrefaciens* by interaction with the c-di-GMP effector, BpfD (Liu et al. 2022). There is compelling evidence that these secondary messengers are key biofilm modulators. During biofilm formation, a high level of intercellular c-di-GMP forces the cells to use a large amount of energy for the production of exopolysaccharides that can subsequently lead to resource depletion and a low cellular metabolic state (Lichtenberg et al. 2022b). The level of c-di-GMP is supposedly a good indicator of the presence of biofilms. The challenge is whether it can be measured direct in clinical biofilms and furthermore, can we expect continuous high levels of c-di-GMP in biofilm cells after prolonged embedment in human tissue?

## Phenotypic features

The phenotypic features of biofilms have been studied extensively to gain insights into how a biofilm functions in different environments. They are crucial for the survival and persistence of biofilms in different harsh environments. All the characteristics presented in this review influence the phenotype of a biofilm. Biofilms often exhibit a high degree of heterogeneity, meaning that different regions within the biofilm can have different populations of bacteria with distinct phenotypes. The phenotypic variations of the individual bacterial cells can be attributed to genetic differences (Hallet 2001), epigenetic modifications (Guespin-Michel 2001, Smits et al. 2006), or environmental cues (Spratt and Lane 2022). This phenotypic heterogeneity enables some bacteria to adopt specialized roles within the biofilm, such as metabolically active cells in surface layers or dormant cells in deeper regions which forms distinctive microenvironments in the spatial organization of a biofilm (Pamp et al. 2008). The heterogeneity of biofilms has predominantly been studied *in vitro*, and it is unclear whether the same spatial differences occur in clinical biofilms; likewise, it is unclear how this differs across various infection sites, bacterial species, and infection durations.

### The metabolic state of a biofilm

Biofilms *per se* are often characterized as inactive/dormant as well as hypoxic or anaerobic. However, this is a dynamic process, as O_2_ is consumed because they have high metabolism during growth; when O_2_ is then depleted, growth will decrease. In the absence of external oxygen sinks, O_2_ will then build up again by diffusion and growth can resume until a steady state is reached, However, *in vivo*, other O_2_ consumers will be present such as PMNs that use O_2_ for their oxygen radical production. This will lead to persistent hypoxic conditions surrounding the biofilms. On the scale of a single biofilm or aggregate, heterogenic metabolic states can develop in very small aggregates (Wessel et al. 2014) where the outer layers of the biofilm are supplied with substrate, which is then depleted towards the inner parts of the biofilm. This can lead to subpopulations displaying different susceptibilities to antibiotics that are influenced by metabolic state (Lichtenberg et al. 2022c). The metabolic state can be manipulated by increasing the supply of substrate, which has been demonstrated by applying hyperbaric oxygen treatment to biofilms which resensitized the biofilm to antibiotics that target actively growing bacteria (Kolpen et al. 2016, 2017, Lerche et al. 2017). A recent publication suggested that single-celled bacteria also displayed low metabolic rates in infections of the lower respiratory tract (Kolpen et al. 2022). Thus, the inactive state is dictated by the environment and may give insight into the phenotype of the bacteria but cannot be used as a defining factor of biofilms.

### Biofilm tolerance

Biofilms possess various mechanisms to increase tolerance to antibiotics and to evade and persist the host immune system. The mechanisms of tolerance towards antibiotics have been thoroughly reviewed elsewhere (see e.g. Van Acker et al. 2014, Ciofu and Tolker-Nielsen 2019, Ciofu et al. 2022), but briefly it can be subdivided into different categories; (i) the physical tolerance, i.e. achieved when penetration is restricted and the antibiotic does not reach all bacteria in the biofilm. (ii) The physiological tolerance, where e.g. slow growth renders the antibiotic target inactive (e.g. protein synthesis). (iii) The transcriptional tolerance, where expression of specific (sets of) genes confers tolerance. This has been argued to include e.g. elevated c-di-GMP levels that lead to upregulation of efflux pumps (Gupta et al. 2014).

To withstand and persist despite a highly activated immune defense some pathogenic bacteria produce various compounds causing necrotic killing of PMNs (Jensen et al. 2007, Löffler et al. 2010). In addition, it has been reported that the size of bacterial aggregates significantly affects the outcome of phagocytosis of *S. aureus, E. coli, P. aeruginosa*, and *S. epidermidis*. Aggregates with a diameter size of 5 um or smaller were successfully phagocytosed by PMNs, while larger aggregates were less likely to be phagocytosed (Alhede et al. 2020b).

The subject of biofilm tolerance is still widely debated but many of the tolerance mechanisms are associated with bacteria residing in dense biofilms while tolerance also occurs in cells not associated with a biofilm. The tolerance of biofilms must be considered the most crucial characteristic in relation to infections.

## Characteristics of clinical biofilms—where are we?

All the characteristics and mechanisms described above, have been shown to contribute to the ‘biofilm’ lifestyle in environmental and *in vitro* grown biofilms. However, the relative importance of each factor is unknown for clinical biofilms. The question is whether they are all present and required to define a clinical biofilm. Microscopy images of tissue sections from patients reveal that clinical biofilms can be organized in very small aggregates consisting of less than 100 cells (Kolpen et al. 2022), but it is unknown whether these small microcolonies show the same characteristics as larger colonies in terms of metabolic state and increased tolerance—characteristics, which are normally used to distinguish biofilms from single cells.

The self-produced EPS matrix has been shown to confer increased tolerance in some settings (Goltermann and Tolker-Nielsen 2017) but on the other hand, the metabolic state of the microorganisms has also been shown to be of major importance (Lopatkin et al. 2019). Thus, an increased antibiotic tolerance may be acquired independently of EPS production. Biofilm infections often have a long-time span with a potential change in characteristics that are not well understood (Cao et al. 2023). Such longitudinal changes are, thus still very difficult to investigate using laboratory- and animal experiments. New technologies, such as MALDI imaging (MALDI mass spectrometry imaging) and scRNA-seq are starting to emerge and being used on clinical samples making it possible to investigate spatial differences in proteomics, metabolomics, and gene expression in and around bacterial communities directly in the infection site. This will undoubtedly yield more knowledge of the clinical biofilm characteristics in the future.

The term ‘biofilm’ can be associated with all the factors described in this review (and more), but despite all the characteristics that have been used to describe biofilms, very few are omnipresent, if any. We are still dependent on visualizing bacteria in the infection to determine if the cells are situated in a biofilm, but even then, the role of nongrowing single cells may be neglected. This questions whether the classification of bacteria according to architecture promotes a better understanding of infections and we argue that for infections, it may be more appropriate to classify bacteria according to treatment response.

## References

[bib1] Alhede M , Alhede M, Qvortrup K et al. The origin of extracellular DNA in bacterial biofilm infections in vivo. Pathog Dis. 2020a;78:ftaa018. 32196074 10.1093/femspd/ftaa018 PMC7150582

[bib2] Alhede M , Lorenz M, Fritz BG et al. Bacterial aggregate size determines phagocytosis efficiency of polymorphonuclear leukocytes. Med Microbiol Immunol. 2020b;209:669–80. 32880037 10.1007/s00430-020-00691-1 PMC7568703

[bib3] Alio I , Gudzuhn M, Perez Garcia P et al. Phenotypic and transcriptomic analyses of seven clinical *Stenotrophomonas maltophilia* isolates identify a small set of shared and commonly regulated genes involved in the biofilm lifestyle. Appl Environ Microb. 2020;86:e02038–20. 10.1128/AEM.02038-20 PMC7688217 33097507

[bib4] Andersen JB , Kragh KN, Hultqvist LD et al. Induction of native c-di-GMP phosphodiesterases leads to dispersal of *Pseudomonas aeruginosa* biofilms. Antimicrob Agents Chemother. 2021;65:e02431–20. 33495218 10.1128/AAC.02431-20 PMC8097489

[bib5] Baraquet C , Harwood CS. Cyclic diguanosine monophosphate represses bacterial flagella synthesis by interacting with the Walker A motif of the enhancer-binding protein FleQ. Proc Natl Acad Sci USA. 2013;110:18478–83. 24167275 10.1073/pnas.1318972110 PMC3832005

[bib6] Bay L , Kragh KN, Eickhardt SR et al. Bacterial aggregates establish at the edges of acute epidermal wounds. Adv Wound Care. 2018;7:105–13. 10.1089/wound.2017.0770 PMC5905854 29675336

[bib7] Bjarnsholt T , Alhede M, Alhede M et al. The in vivo biofilm. Trends Microbiol. 2013;21:466–74. 23827084 10.1016/j.tim.2013.06.002

[bib8] Bjarnsholt T , Whiteley M, Rumbaugh KP et al. The importance of understanding the infectious microenvironment. Lancet Infect Dis. 2022;22:e88–92. 34506737 10.1016/S1473-3099(21)00122-5 PMC9190128

[bib9] Borlee BR , Goldman AD, Murakami K et al. *Pseudomonas aeruginosa* uses a cyclic-di-GMP-regulated adhesin to reinforce the biofilm extracellular matrix. Mol Microbiol. 2010;75:827–42. 20088866 10.1111/j.1365-2958.2009.06991.x PMC2847200

[bib10] Brennan MA , Rosenthal AZ. Single-cell RNA sequencing elucidates the structure and organization of microbial communities. Front Microbiol. 2021;12:713128. 34367118 10.3389/fmicb.2021.713128 PMC8334356

[bib11] Cao P , Fleming D, Moustafa DA et al. A *Pseudomonas aeruginosa* small RNA regulates chronic and acute infection. Nature. 2023;618. 10.1038/s41586-023-06111-7. PMC10247376 37225987

[bib12] Chenicheri S , Usha R, Ramachandran R et al. Insight into oral biofilm: primary, secondary and residual caries and phyto-challenged solutions. Open Dent J. 2017;11:312–33. 28839480 10.2174/1874210601711010312 PMC5543615

[bib13] Chua SL , Liu Y, Yam JK et al. Dispersed cells represent a distinct stage in the transition from bacterial biofilm to planktonic lifestyles. Nat Commun. 2014;5:4462. 25042103 10.1038/ncomms5462

[bib14] Ciofu O , Moser C, Jensen PO et al. Tolerance and resistance of microbial biofilms. Nat Rev Micro. 2022;20. 10.1038/s41579-022-00682-4. 35115704

[bib15] Ciofu O , Tolker-Nielsen T. Tolerance and resistance of *Pseudomonas aeruginosa* biofilms to antimicrobial agents—how *P. aeruginosa* can escape antibiotics. Front Microbiol. 2019;10:114–31. 31130925 10.3389/fmicb.2019.00913 PMC6509751

[bib16] Coenye T. Response of sessile cells to stress: from changes in gene expression to phenotypic adaptation. FEMS Immunol Med Microbiol. 2010;59:239–52. 20482621 10.1111/j.1574-695X.2010.00682.x

[bib17] Collins AJ , Smith TJ, Sondermann H et al. From input to output: the lap/c-di-GMP biofilm regulatory circuit. Annu Rev Microbiol. 2020;74:607–31. 32689917 10.1146/annurev-micro-011520-094214 PMC8966053

[bib18] Cornforth DM , Dees JL, Ibberson CB et al. *Pseudomonas aeruginosa* transcriptome during human infection. Proc Natl Acad Sci USA. 2018;115:E5125–E34. 29760087 10.1073/pnas.1717525115 PMC5984494

[bib19] Cornforth DM , Diggle FL, Melvin JA et al. Quantitative framework for model evaluation in microbiology research using *Pseudomonas aeruginosa* and cystic fibrosis infection as a test case. mBio. 2020;11:e03042–19. 31937646 10.1128/mBio.03042-19 PMC6960289

[bib20] Costerton JW , Stewart PS, Greenberg EP. Bacterial biofilms: a common cause of persistent infections. Science. 1999;284:1318–22. 10334980 10.1126/science.284.5418.1318

[bib21] Cowan SE , Gilbert E, Liepmann D et al. Commensal interactions in a dual-species biofilm exposed to mixed organic compounds. Appl Environ Microb. 2000;66:4481–5. 10.1128/aem.66.10.4481-4485.2000 PMC92328 11010902

[bib22] Dahlstrom KM , O'Toole GA. A symphony of cyclases: specificity in diguanylate cyclase signaling. Annu Rev Microbiol. 2017;71:179–95. 28645224 10.1146/annurev-micro-090816-093325 PMC5936083

[bib23] Dar D , Dar N, Cai L et al. Spatial transcriptomics of planktonic and sessile bacterial populations at single-cell resolution. Science. 2021;373:eabi4882. 34385369 10.1126/science.abi4882 PMC8454218

[bib24] Dotsch A , Eckweiler D, Schniederjans M et al. The *Pseudomonas aeruginosa* transcriptome in planktonic cultures and static biofilms using RNA sequencing. PLoS One. 2012;7:e31092. 22319605 10.1371/journal.pone.0031092 PMC3272035

[bib25] Dotsch A , Schniederjans M, Khaledi A et al. The *Pseudomonas aeruginosa* transcriptional landscape is shaped by environmental heterogeneity and genetic variation. mBio. 2015;6:e00749. 26126853 10.1128/mBio.00749-15 PMC4488947

[bib26] Fazli M , Almblad H, Rybtke ML et al. Regulation of biofilm formation in *Pseudomonas* and *Burkholderia* species. Environ Microbiol. 2014;16:1961–81. 24592823 10.1111/1462-2920.12448

[bib27] Flemming HC , van Hullebusch ED, Neu TR et al. The biofilm matrix: multitasking in a shared space. Nat Rev Micro. 2023;21:70–86. 10.1038/s41579-022-00791-0 36127518

[bib28] Flemming HC , Wuertz S. Bacteria and archaea on Earth and their abundance in biofilms. Nat Rev Micro. 2019;17:247–60. 10.1038/s41579-019-0158-9 30760902

[bib29] Goltermann L , Tolker-Nielsen T. Importance of the exopolysaccharide matrix in antimicrobial tolerance of *Pseudomonas aeruginosa* aggregates. Antimicrob Agents Chemother. 2017;61:e02696–16. 28137803 10.1128/AAC.02696-16 PMC5365683

[bib30] Guespin-Michel J. Epigenesis–a request for information on loss of adaptive phenotypes. Microbiology. 2001;147:252–3. 11158342 10.1099/00221287-147-2-252

[bib31] Guo R , Lu H. Targeted metabolomics revealed the regulatory role of Manganese on small-molecule metabolism of biofilm formation in *Escherichia coli*. J Anal Test. 2020;4:226–37.

[bib32] Guo R , Luo X, Liu J et al. Mass spectrometry based targeted metabolomics precisely characterized new functional metabolites that regulate biofilm formation in *Escherichia coli*. Anal Chim Acta. 2021;1145:26–36. 33453877 10.1016/j.aca.2020.12.021

[bib33] Gupta K , Liao J, Petrova OE et al. Elevated levels of the second messenger c-di-GMP contribute to antimicrobial resistance of *Pseudomonas aeruginosa*. Mol Microbiol. 2014;92:488–506. 24655293 10.1111/mmi.12587 PMC4029167

[bib35] Hall-Stoodley L , Costerton JW, Stoodley P. Bacterial biofilms: from the natural environment to infectious diseases. Nat Rev Microbiol. 2004;2:95–108. 15040259 10.1038/nrmicro821

[bib34] Hallet B. Playing Dr Jekyll and Mr Hyde: combined mechanisms of phase variation in bacteria. Curr Opin Microbiol. 2001;4:570–81. 11587935 10.1016/s1369-5274(00)00253-8

[bib36] Harrington NE , Littler JL, Harrison F. Transcriptome analysis of *Pseudomonas aeruginosa* biofilm infection in an ex vivo pig model of the cystic fibrosis lung. Appl Environ Microb. 2022;88:e0178921. 10.1128/aem.01789-21 PMC8824274 34878811

[bib37] Heacock-Kang Y , Sun Z, Zarzycki-Siek J et al. Spatial transcriptomes within the *Pseudomonas aeruginosa* biofilm architecture. Mol Microbiol. 2017;106:976–85. 29030956 10.1111/mmi.13863 PMC5720903

[bib38] Hengge R. Principles of c-di-GMP signalling in bacteria. Nat Rev Micro. 2009;7:263–73. 10.1038/nrmicro2109 19287449

[bib39] Henrici AT. Studies of freshwater bacteria: I. A direct microscopic technique. J Bacteriol. 1933;25:277–87. 16559616 10.1128/jb.25.3.277-287.1933 PMC533461

[bib40] Hoiby N. *Pseudomonas aeruginosa* infection in cystic fibrosis. Diagnostic and prognostic significance of *Pseudomonas aeruginosa* precipitins determined by means of crossed immunoelectrophoresis. A survey. Acta Pathol Microbiol Scand Suppl. 1977;58:1–96. 411327

[bib41] Homberger C , Hayward RJ, Barquist L et al. Improved bacterial single-cell RNA-seq through automated MATQ-seq and Cas9-based removal of rRNA reads. mBio. 2023;14:e0355722. 36880749 10.1128/mbio.03557-22 PMC10127585

[bib42] Ibberson CB , Whiteley M. The Staphylococcus aureus transcriptome during cystic fibrosis lung infection. mBio. 2019;10:e02774–19. 31744924 10.1128/mBio.02774-19 PMC6867902

[bib43] Ito A , May T, Taniuchi A et al. Localized expression profiles of rpoS in *Escherichia coli* biofilms. Biotechnol Bioeng. 2009;103:975–83. 19288441 10.1002/bit.22305

[bib44] Jennings LK , Dreifus JE, Reichhardt C et al. *Pseudomonas aeruginosa* aggregates in cystic fibrosis sputum produce exopolysaccharides that likely impede current therapies. Cell Rep. 2021;34:108782. 33626358 10.1016/j.celrep.2021.108782 PMC7958924

[bib45] Jensen PO , Bjarnsholt T, Phipps R et al. Rapid necrotic killing of polymorphonuclear leukocytes is caused by quorum-sensing-controlled production of rhamnolipid by *Pseudomonas aeruginosa*. Microbiology. 2007;153:1329–38. 17464047 10.1099/mic.0.2006/003863-0

[bib46] Kirketerp-Moller K , Jensen PO, Fazli M et al. Distribution, organization, and ecology of bacteria in chronic wounds. J Clin Microbiol. 2008;46:2717–22. 18508940 10.1128/JCM.00501-08 PMC2519454

[bib47] Kirketerp-Møller K , Stewart PS, Bjarnsholt T. The zone model: a conceptual model for understanding the microenvironment of chronic wound infection. Wound Repair Regen. 2020;28:593–9. 32529778 10.1111/wrr.12841 PMC7540265

[bib48] Kolpen M , Kragh KN, Enciso JB et al. Bacterial biofilms predominate in both acute and chronic human lung infections. Thorax. 2022;77:1015–22. 35017313 10.1136/thoraxjnl-2021-217576 PMC9510407

[bib49] Kolpen M , Lerche CJ, Kragh KN et al. Hyperbaric oxygen sensitizes anoxic *Pseudomonas aeruginosa* biofilm to ciprofloxacin. Antimicrob Agents Chemother. 2017;61:e01024–17. 28874373 10.1128/AAC.01024-17 PMC5655102

[bib50] Kolpen M , Mousavi N, Sams T et al. Reinforcement of the bactericidal effect of ciprofloxacin on *Pseudomonas aeruginosa* biofilm by hyperbaric oxygen treatment. Int J Antimicrob Agents. 2016;47:163–7. 26774522 10.1016/j.ijantimicag.2015.12.005

[bib103_1693810109631] Kragh KN , Tolker-Nielsen T, Lichtenberg M. The non-attached biofilm aggregate. Commun Biol. 2023. 10.1038/s42003-023-05281-4. PMC10474055 37658117

[bib51] Kuchina A , Brettner LM, Paleologu L et al. Microbial single-cell RNA sequencing by split-pool barcoding. Science. 2021;371:371. 33335020 10.1126/science.aba5257 PMC8269303

[bib52] Lawrence JR , Korber DR, Hoyle BD et al. Optical sectioning of microbial biofilms. J Bacteriol. 1991;173:6558–67. 1917879 10.1128/jb.173.20.6558-6567.1991 PMC208993

[bib53] Le Masters T , Johnson S, Jeraldo PR et al. Comparative transcriptomic analysis of *Staphylococcus aureus* associated with periprosthetic joint infection under in vivo and in vitro conditions. J Mol Diagn. 2021;23:986–99. 34098085 10.1016/j.jmoldx.2021.05.011 PMC8351120

[bib54] Lenz AP , Williamson KS, Pitts B et al. Localized gene expression in *Pseudomonas aeruginosa* biofilms. Appl Environ Microb. 2008;74:4463–71. 10.1128/AEM.00710-08 PMC2493172 18487401

[bib55] Lerche CJ , Christophersen LJ, Kolpen M et al. Hyperbaric oxygen therapy augments tobramycin efficacy in experimental *Staphylococcus aureus* endocarditis. Int J Antimicrob Agents. 2017;50:406–12. 28669832 10.1016/j.ijantimicag.2017.04.025

[bib56] Lewin GR , Kapur A, Cornforth DM et al. Application of a quantitative framework to improve the accuracy of a bacterial infection model. Proc Natl Acad Sci USA. 2023;120:e2221542120. 37126703 10.1073/pnas.2221542120 PMC10175807

[bib57] Lichtenberg M , Jakobsen TH, Kuhl M et al. The structure-function relationship of Pseudomonas aeruginosa in infections and its influence on the microenvironment. FEMS Microbiol Rev. 2022a;46:fuac018. 35472245 10.1093/femsre/fuac018 PMC9438473

[bib58] Lichtenberg M , Kragh KN, Fritz B et al. Cyclic-di-GMP signaling controls metabolic activity in *Pseudomonas aeruginosa*. Cell Rep. 2022b;41:111515. 36260996 10.1016/j.celrep.2022.111515

[bib59] Lichtenberg M , Kvich L, Larsen SLB et al. Inoculum concentration influences *Pseudomonas aeruginosa* phenotype and biofilm architecture. Microbiol Spectr. 2022c;10:e0313122. 36354337 10.1128/spectrum.03131-22 PMC9769529

[bib60] Liu C , Sun D, Liu J et al. cAMP and c-di-GMP synergistically support biofilm maintenance through the direct interaction of their effectors. Nat Commun. 2022;13:1493. 35315431 10.1038/s41467-022-29240-5 PMC8938473

[bib61] Liu C , Sun D, Zhu J et al. The regulation of bacterial biofilm formation by cAMP-CRP: a mini-review. Front Microbiol. 2020;11:802. 32528421 10.3389/fmicb.2020.00802 PMC7247823

[bib62] Livingston J , Spero MA, Lonergan ZR et al. Visualization of mRNA expression in *Pseudomonas aeruginosa* aggregates reveals spatial patterns of fermentative and denitrifying metabolism. Appl Environ Microb. 2022;88:e0043922. 10.1128/aem.00439-22 PMC9195945 35586988

[bib63] Löffler B , Hussain M, Grundmeier M et al. *Staphylococcus aureus* Panton-Valentine leukocidin is a very potent cytotoxic factor for human neutrophils. PLoS Pathog. 2010;6:e1000715. 20072612 10.1371/journal.ppat.1000715 PMC2798753

[bib64] Lopatkin AJ , Stokes JM, Zheng EJ et al. Bacterial metabolic state more accurately predicts antibiotic lethality than growth rate. Nat Microbiol. 2019;4:2109–17. 31451773 10.1038/s41564-019-0536-0 PMC6879803

[bib65] Lu H , Que Y, Wu X et al. Metabolomics deciphered metabolic reprogramming required for biofilm formation. Sci Rep. 2019;9:13160. 31511592 10.1038/s41598-019-49603-1 PMC6739361

[bib66] Martinez-Mendez R , Camacho-Hernandez DA, Sulvaran-Guel E et al. A trigger phosphodiesterase modulates the global c-di-GMP pool, motility, and biofilm formation in *Vibrio parahaemolyticus*. J Bacteriol. 2021;203:e0004621. 33846117 10.1128/JB.00046-21 PMC8316121

[bib67] McBain AJ. Chapter 4 in vitro biofilm models: an overview. Adv Appl Microbiol. 2009;69:99–132. 19729092 10.1016/S0065-2164(09)69004-3

[bib68] McCoy WF , Bryers JD, Robbins J et al. Observations of fouling biofilm formation. Can J Microbiol. 1981;27:910–7. 7306879 10.1139/m81-143

[bib69] McNulty R , Sritharan D, Pahng SH et al. Probe-based bacterial single-cell RNA sequencing predicts toxin regulation. Nat Microbiol. 2023;8:934–45. 37012420 10.1038/s41564-023-01348-4 PMC10159851

[bib70] Pabst B , Pitts B, Lauchnor E et al. Gel-entrapped *Staphylococcus aureus* bacteria as models of biofilm infection exhibit growth in dense aggregates, oxygen limitation, antibiotic tolerance, and heterogeneous gene expression. Antimicrob Agents Chemother. 2016;60:6294–301. 27503656 10.1128/AAC.01336-16 PMC5038234

[bib71] Pamp SJ , Gjermansen M, Johansen HK et al. Tolerance to the antimicrobial peptide colistin in *Pseudomonas aeruginosa* biofilms is linked to metabolically active cells, and depends on the pmr and mexAB-oprM genes. Mol Microbiol. 2008;68:223–40. 18312276 10.1111/j.1365-2958.2008.06152.x

[bib72] Pamp SJ , Tolker-Nielsen T. Multiple roles of biosurfactants in structural biofilm development by *Pseudomonas aeruginosa*. J Bacteriol. 2007;189:2531–9. 17220224 10.1128/JB.01515-06 PMC1899385

[bib73] Perez-Osorio AC , Williamson KS, Franklin MJ. Heterogeneous rpoS and rhlR mRNA levels and 16S rRNA/rDNA (rRNA gene) ratios within *Pseudomonas aeruginosa* biofilms, sampled by laser capture microdissection. J Bacteriol. 2010;192:2991–3000. 20348255 10.1128/JB.01598-09 PMC2901698

[bib74] Pettygrove BA , Kratofil RM, Alhede M et al. Delayed neutrophil recruitment allows nascent *Staphylococcus aureus* biofilm formation and immune evasion. Biomaterials. 2021;275:120775. 34243039 10.1016/j.biomaterials.2021.120775 PMC8325624

[bib75] Reysenbach A-L , Cady SL. Microbiology of ancient and modern hydrothermal systems. Trends Microbiol. 2001;9:79–86. 11173247 10.1016/s0966-842x(00)01921-1

[bib76] Ryall B , Eydallin G, Ferenci T. Culture history and population heterogeneity as determinants of bacterial adaptation: the adaptomics of a single environmental transition. Microbiol Mol Biol Rev. 2012;76:597–625. 22933562 10.1128/MMBR.05028-11 PMC3429624

[bib77] Sauer K , Stoodley P, Goeres DM et al. The biofilm life cycle: expanding the conceptual model of biofilm formation. Nat Rev Micro. 2022;20:608–20. 10.1038/s41579-022-00767-0 PMC9841534 35922483

[bib78] Schembri MA , Kjaergaard K, Klemm P. Global gene expression in *Escherichia coli* biofilms. Mol Microbiol. 2003;48:253–67. 12657059 10.1046/j.1365-2958.2003.03432.x

[bib79] Schleheck D , Barraud N, Klebensberger J et al. *Pseudomonas aeruginosa* PAO1 preferentially grows as aggregates in liquid batch cultures and disperses upon starvation. PLoS ONE. 2009;4:e5513. 19436737 10.1371/journal.pone.0005513 PMC2677461

[bib80] Smits WK , Kuipers OP, Veening JW. Phenotypic variation in bacteria: the role of feedback regulation. Nat Rev Micro. 2006;4:259–71. 10.1038/nrmicro1381 16541134

[bib81] Sohm JA , Edwards BR, Wilson BG et al. Constitutive extracellular polysaccharide (EPS) production by specific isolates of *Crocosphaera watsonii*. Front Microbiol. 2011;2:229. 22110469 10.3389/fmicb.2011.00229 PMC3215947

[bib82] Spratt MR , Lane K. Navigating environmental transitions: the role of phenotypic variation in bacterial responses. mBio. 2022;13:e0221222. 36259726 10.1128/mbio.02212-22 PMC9765552

[bib83] Stewart PS , Franklin MJ. Physiological heterogeneity in biofilms. Nat Rev Micro. 2008;6:199–210. 10.1038/nrmicro1838 18264116

[bib84] Stewart PS , White B, Boegli L et al. Conceptual model of biofilm antibiotic tolerance that integrates phenomena of diffusion, metabolism, gene expression, and physiology. J Bacteriol. 2019;201:2125–24. 10.1128/JB.00307-19 PMC6805107 31501280

[bib85] Stewart PS , Zhang T, Xu R et al. Reaction-diffusion theory explains hypoxia and heterogeneous growth within microbial biofilms associated with chronic infections. npj Biofilms Microbiomes. 2016;2:16012. 28721248 10.1038/npjbiofilms.2016.12 PMC5515263

[bib86] Stewart PS. Diffusion in biofilms. J Bacteriol. 2003;185:1485–91. 12591863 10.1128/JB.185.5.1485-1491.2003 PMC148055

[bib87] Thoming JG , Tomasch J, Preusse M et al. Parallel evolutionary paths to produce more than one *Pseudomonas aeruginosa* biofilm phenotype. npj Biofilms Microbiomes. 2020;6:2. 31934344 10.1038/s41522-019-0113-6 PMC6954232

[bib88] Toliopoulos C , Giaouris E. Marked inter-strain heterogeneity in the differential expression of some key stress response and virulence-related genes between planktonic and biofilm cells in *Listeria monocytogenes*. Int J Food Microbiol. 2023;390:110136. 36807004 10.1016/j.ijfoodmicro.2023.110136

[bib89] Van Acker H , Van Dijck P, Coenye T. Molecular mechanisms of antimicrobial tolerance and resistance in bacterial and fungal biofilms. Trends Microbiol. 2014;22:326–33. 24598086 10.1016/j.tim.2014.02.001

[bib90] Vlaeminck J , Lin Q, Xavier BB et al. The dynamic transcriptome during maturation of biofilms formed by methicillin-resistant *Staphylococcus aureus*. Front Microbiol. 2022;13:882346. 35966712 10.3389/fmicb.2022.882346 PMC9366926

[bib92] Wang T-Y , Guo R, Hu L-L et al. Mass spectrometry-based targeted metabolomics revealed the regulatory roles of magnesium on biofilm formation in *Escherichia coli* by targeting functional metabolites. J Anal Test. 2022a;6:89–97.

[bib93] Wang Y , Sun L, Hu L et al. Adhesion and kinetics of biofilm formation and related gene expression of *Listeria monocytogenes* in response to nutritional stress. Food Res Int. 2022b;156:111143. 35651015 10.1016/j.foodres.2022.111143

[bib91] Wang T , Shen P, He Y et al. Spatial transcriptome uncovers rich coordination of metabolism in *E. coli* K12 biofilm. Nat Chem Biol. 2023;19. 10.1038/s41589-023-01282-w. 37055614

[bib94] Weismann U , Choi IS, Dombrowski E-M. Fundamentals of Biological Wastewater Treatment. Hoboken: John Wiley & Sons, 2007.

[bib95] Wessel AK , Arshad TA, Fitzpatrick M et al. Oxygen limitation within a bacterial aggregate. mBio. 2014;5:e00992. 24736225 10.1128/mBio.00992-14 PMC3994514

[bib96] Whiteley M , Bangera MG, Bumgarner RE et al. Gene expression in *Pseudomonas aeruginosa* biofilms. Nature. 2001;413:860–4. 11677611 10.1038/35101627

[bib97] Wille J , Teirlinck E, Sass A et al. Does the mode of dispersion determine the properties of dispersed *Pseudomonas aerinosa* biofilm cells?. Int J Antimicrob Agents. 2020;56:106194. 33039591 10.1016/j.ijantimicag.2020.106194

[bib98] Williamson KS , Richards LA, Perez-Osorio AC et al. Heterogeneity in *Pseudomonas aeruginosa* biofilms includes expression of ribosome hibernation factors in the antibiotic-tolerant subpopulation and hypoxia-induced stress response in the metabolically active population. J Bacteriol. 2012;194:2062–73. 22343293 10.1128/JB.00022-12 PMC3318454

[bib99] Wingender J , Neu TR, Flemming H-C. What are bacterial extracellular polymeric substances?. In: Wingender J, Neu TR, Flemming H-C (eds), Microbial Extracellular Polymeric Substances: Characterization, Structure and Function, Berlin, Heidelberg: Springer, 1999, 1–19. 10.1007/978-3-642-60147-7_1.

[bib100] Xu Y , Maltesen RG, Larsen LH et al. In vivo gene expression in a *Staphylococcus aureus* prosthetic joint infection characterized by RNA sequencing and metabolomics: a pilot study. BMC Microbiol. 2016;16:80. 27150914 10.1186/s12866-016-0695-6 PMC4858865

[bib101] Zheng L , Zhang X, Lu Z et al. Transcriptome sequencing reveals the difference in the expression of biofilm and planktonic cells between two strains of *Salmonella* Typhimurium. Biofilm. 2022;4:100086. 36254114 10.1016/j.bioflm.2022.100086 PMC9568869

[bib102] Zobell CE , Allen EC. The significance of marine bacteria in the fouling of submerged surfaces. J Bacteriol. 1935;29:239–51. 16559784 10.1128/jb.29.3.239-251.1935 PMC543592

